# Streptococcal adhesin SspA/B analogue peptide inhibits adherence and impacts biofilm formation of *Streptococcus mutans*

**DOI:** 10.1371/journal.pone.0175483

**Published:** 2017-04-10

**Authors:** Tatsuro Ito, Takahiro Ichinosawa, Takehiko Shimizu

**Affiliations:** 1 Department of Pediatric Dentistry, Nihon University School of Dentistry at Matsudo, Chiba, Japan; 2 Nihon University Research Institute of Oral Science, Chiba, Japan; LSU Health Sciences Center School of Dentistry, UNITED STATES

## Abstract

*Streptococcus mutans*, the major causative agent of dental caries, adheres to tooth surfaces via the host salivary glycoprotein-340 (gp340). This adherence can be competitively inhibited by peptides derived from the SspA/B adhesins of *Streptococcus gordonii*, a human commensal microbe that competes for the same binding sites. Ssp(A4K-A11K), a double-lysine substituted SspA/B peptide analogue, has been shown to exhibit superior *in vitro* binding affinity for a gp340-derived peptide (SRCRP2), suggesting that Ssp(A4K-A11K) may be of clinical interest. In the present work, we tested the inhibitory effects of Ssp(A4K-A11K) on adherence and biofilm formation of *S*. *mutans* by reconstructing an artificial oral environment using saliva-coated polystyrene plates and hydroxyapatite disks. Bacterial adherence (adherence period: 1 h) was assessed by an enzyme-linked immunosorbent assay using biotinylated bacterial cells. Biofilm formation (periods: 8, 11, or 14 h) was assessed by staining and imaging of the sessile cells, or by recovering biofilm cells and plating for cell counts. The pH values of the culture media were measured as a biofilm acidogenicity indicator. Bactericidality was measured by loss of optical density during culturing in the presence of the peptide. We observed that 650 μM Ssp(A4K-A11K) significantly inhibited adherence of *S*. *mutans* to saliva-coated polystyrene; a similar effect was seen on bacterial affinity for SRCRP2. Ssp(A4K-A11K) had lesser effects on the adherence of commensal streptococci. Pretreatment of polystyrene and hydroxyapatite with 650 μM Ssp(A4K-A11K) significantly attenuated biofilm formation, whether tested with glucose- or sucrose-containing media. The SspA/B peptide’s activity did not reflect bactericidality. Strikingly, pH in Ssp-treated 8-h (6.8 ± 0.06) and 11-h (5.5 ± 0.06) biofilms showed higher values than the critical pH. Thus, Ssp(A4K-A11K) acts by inhibiting bacterial adherence and cariogrnic biofilm formation. We further consider these results in the context of the safety, specificity, and stability properties of the Ssp(A4K-A11K) peptide.

## Introduction

*Streptococcus mutans* is the major causative agent of dental caries and plays an important role in cariogenic biofilm formation [1, 2]. The cell surface adhesin of *S*. *mutans*, variously designated as AgI/II (reviewed by [3]), PAc [4], P1 [5], B [6], and MSL-1 [7], mediates adhesion of the organism to salivary acquired enamel pellicles along with dental plaque biofilm development [8]. AgI/II of *S*. *mutans* interacts specifically with the innate immunity scavenger receptor glycoprotein-340 (gp340) [9] present in saliva. When gp340 is adsorbed onto a tooth surface, this protein serves as a receptor for streptococcal adherence, including that of *S*. *mutans* [10, 11].

*Streptococcus gordonii* is a primary colonizer of dental plaque biofilms and also interacts with salivary gp340 via the adhesins SspA and SspB (SspA/B) [7, 12]. The adhesins SspA/B of *S*. *gordonii* have extensive homology with AgI/II of *S*. *mutans*; hence, *S*. *gordonii* competes with *S*. *mutans* for binding to the same array of available host receptors on a pellicle layer [13–15]. Recently, an SspB-derived analogous peptide, SspB(390-T400K-402), that possesses high-affinity binding to salivary gp340 peptide SRCRP2 [16] was shown to competitively block *S*. *mutans* adhesion to experimental pellicles, thereby inhibiting biofilm formation [17]. However, although various types of agents with anti-microbial activity against *S*. *mutans* have been reported [17–20], safety, specificity, and stability of the agents remain a concern. Moreover, limited studies have been performed to evaluate the impact of such agents on resident oral microflora.

The importance of ion-pairing interactions (lysine and negatively charged functional groups) has been revealed for protein stabilization [21], suggesting that lysine is likely the core residue for binding pathogen proteins to negatively charged surfaces on salivary components. Ssp(A4K-A11K), an SspA/B analogue peptide [11], corresponds to a consensus sequence of SspA/B in which substitutions with lysine (at positions 4 and 11) facilitate durable interactions with SRCRP2. Koba et al. [22] have demonstrated that Ssp(A4K-A11K) has the highest binding activity to the salivary components and to SRCRP2 compared with several analogous SspA/B peptides. Considered together, these results suggest that Ssp(A4K-A11K) may be safer, more specific, and more stable in inhibiting *S*. *mutans* biofilm formation than native SspA/B peptides.

Here we conclusively demonstrate the inhibitory effects of Ssp(A4K-A11K) on adherence and biofilm formation of *S*. *mutans* by reconstructing an artificial oral environment using saliva-coated polystyrene plates and hydroxyapatite disks.

## Materials and methods

### Bacterial culture

*Streptococcus mutans* MT 8148 known as a proven virulent cariogenic pathogen, *Streptococcus gordonii* DL1, *Streptococcus mitis* ATCC 6249, and *Streptococcus salivarius* ATCC 9759 (kindly provided by Dr. Hidenobu Senpuku, National Institute of Infectious Diseases) were maintained in brain heart infusion (BHI) broth under anaerobic conditions (10% CO_2_, 10% H_2_, and 80% N_2_).

### Peptide synthesis

The Ssp(A4K-A11K) peptide, DYQKKLAAYQKEL, was designed by substitution of K (lysine) for A (alanine) at position 4 and position 11 in the consensus sequence (DYQAKLAAYQAEL) of SspA/B peptides [22]. The SRCRP2 peptide, QGRVEVLYRGSWGTVC, is a sub-sequence of salivary gp340 [23] and was used as a salivary component. Peptides were synthesized at 95% purity by Scrum, Inc. (Tokyo, Japan), and suspended in sterile distilled water (DW) at the desired concentration immediately before use.

### Human saliva collection

As described previously [24], saliva samples were collected from volunteers in good oral health, with saliva collection performed after stimulation by chewing of paraffin gum. The experimental protocol was reviewed and approved by the Nihon University Institutional Review Board (EC14-001). All subjects’ rights were protected and informed consent was granted in writing. Volunteers had refrained from eating, drinking, and brushing for at least 2 h prior to collection. Saliva was placed in ice-chilled sterile bottles for 5 min, followed by centrifugation at 10,000 × *g* for 10 min at 4°C in order to remove cellular debris. Supernatants were filter sterilized through a 0.22-μm Millex-GP filter (Merck Millipore, Bedford, MA, USA). After filtration, samples were pooled and stored at −20°C until use.

### Bacterial adhesion assay

To detect inhibitory effects of Ssp(A4K-A11K) in a bacterial assay of the adhesion of streptococci to experimental pellicles, *S*. *mutans*, *S*. *gordonii*, *S*, *mitis*, and *S*. *salivarius* were biotinylated [25] for use in an enzyme-linked immunosorbent assay (ELISA). Cultures of streptococci were washed with phosphate-buffered saline (PBS). The bacterial cells were then biotinylated by incubation with NHS-LC-Biotin (Pierce) at 100 μg/ml for 1 h at room temperature. After washing with PBS, the bacterial density was adjusted to an optical density at 600 nm of 0.4. Ninety-six-well ELISA plates (Sumitomo Bakelite, Tokyo, Japan) were then coated with 100 μl per well of sterile whole saliva or salivary agglutinin peptide SRCRP2 (200 μg/ml) for 1 h at 4°C. Next, 100 μl of SspA/B peptides (A4K-A11K or consensus sequence peptide) were added to saliva- or SRCRP2-coated wells and incubated for 1 h at 37°C. After two washes with PBS containing 0.1% Tween 20 (PBST), 100 μl of biotinylated bacterial cells were added and subsequently incubated for 1 h at 37°C. The wells were then incubated for 1 h at 37°C with alkaline phosphatase-conjugated streptavidin (Invitrogen, Carlsbad, CA, USA) at a dilution of 1:1,000. Bacteria that adhered to saliva or SRCRP2 then were detected by chromogenic development using para-nitrophenyl phosphate as the alkaline phosphatase substrate. After development, absorbance at 405 nm was measured and compared with control (non-treatment of saliva).

### Peptide binding assay

Binding activity of Ssp(A4K-A11K) peptide to SRCRP2 was detected by sandwich assay [11]. We sandwiched SRCRP2 between biotinylated and non-biotinylated Ssp(A4K-A11K) peptides. Briefly, 96-well ELISA plates were coated with Ssp(A4K-A11K) overnight at 4°C. After overnight incubation, SRCRP2 or bovine serum albumin (BSA: a control) was added (100 μl per well) and incubated at 4°C for 1 h. Biotinylated Ssp(A4K-A11K) peptide (650 μM) in 100 μl of sterile DW was then applied to the wells; *i*.*e*., SRCRP2 was placed between Ssp(A4K-A11K) and biotinylated Ssp(A4K-A11K). Reactions were detected using the same ELISA protocol as mentioned above.

### Bactericidal assay

*S*. *mutans* was grown at 37°C for 18 h under anaerobic conditions. After three times washing with sterile PBS, harvested bacteria were suspended in BHI and adjusted to 3.8 × 10^8^ colony-forming units (CFUs) in a 3-ml bacterial suspension. Six hundred and fifty μM Ssp(A4K-A11K) peptide or 0.04% chlorhexidine (CHX) were added to separate bacterial cultures. The mix suspensions were anaerobically incubated for 16 or 22 h. The bacterial densities at 600 nm were measured using a spectrophotometer (mini photo 518R; Taitec, Co., Saitama, Japan) and compared with those of control suspensions (no peptide or CHX).

### Biofilm formation assay on polystyrene microtiter plates

Biofilm formation by *S*. *mutans* was assayed using the method of Okuda et al. [17] with some modifications. This assay used 96-well (flat-bottom) polystyrene microtiter plates (Sumitomo Bakelite, Tokyo, Japan) as convenient and sterile abiotic surfaces for studying bacterial biofilm formation. In brief, the wells were coated with sterile whole saliva at 4°C overnight and then 100 μl of the analogue peptide solution (650 μM in DW) was added to each well and allowed to incubate for 1 h at 37°C. After three washes with sterile PBS, a suspension of *S*. *mutans* [2.5 × 10^6^ CFU in 160 μl tryptic soy broth without dextrose supplemented with 0.25% glucose (TSBG) or with 0.25% sucrose (TSBS)] was distributed to each well and allowed to grow for 8, 11, or 14 h at 37°C under anaerobic conditions. To investigate the impact of Ssp analogue to later stage biofilm, *S*. *mutans* was grown in TSBS for 48 h, 72 h, and 1 week. The pH of spent culture media was measured using a standard pH electrode (HORIBA, Ltd., Kyoto, Japan). After incubation for the indicated time, biofilms formed on the wells were rinsed with sterile PBS, air-dried, stained with safranin for 15 min, and washed with DW to remove excess dye. The biofilm mass was quantified by measuring absorbance at 492 nm.

### Biofilm evaluation on saliva-coated hydroxyapatite disks

To further assess effects on biofilm formation, we evaluated biofilms on hydroxyapatite (HA) disks by the method of Ahn et al. [26] with some modifications. Briefly, autoclaved HA disks (10.0-mm diameter and 2.0-mm thickness; HOYA Technosurgical, Tokyo, Japan) were placed into 24-well microtiter plates and were coated with sterile whole saliva (s-HA) at 4°C overnight. After removing the saliva, 300 μl of Ssp(A4K-A11K) peptide solution (650 μM in DW) was added to each well and incubated for 1 h at 37°C. After two washes with sterile PBS, each well received 50 μl of *S*. *mutans* cell suspension (6.3 × 10^6^ CFU/ml) along with 450 μl of TSBS, and the plates were incubated anaerobically for 8, 11, or 14 h at 37°C. The culture medium, including planktonic cells and loosely bound cells, was removed, and the disks were rinsed with sterile PBS. Each disk was transferred to a conical tube containing 3 ml PBS. The adherent bacteria were detached by sonication (Sonicator, Ohtake, Co., Tokyo, Japan) using four 30-s pulses at 25 W with three 30-s intermittent cooling stages in an ice-chilled box. The cell suspensions were serially diluted and plated on Mitis Salivarius Bacitracin (MSB) agar, followed by a 2 day-incubation at 37°C. The numbers of bacterial colonies were counted and expressed as CFU/ml.

### Statistical analyses

Data are expressed as means with standard deviations. GraphPad Prism version 5.0 d for Mac OS X (GraphPad Software, San Diego, CA) was used to assess significance. The statistical significance of differences between two groups was determined by unpaired *t*-tests. When the samples had unequal variances, unpaired *t*-tests with Welch’s correction were used. For comparisons between multiple groups, one-way analysis of variance (ANOVA) and post-hoc Tukey-Kramer tests were used. *P*-values less than 0.01 or 0.05 were considered to be statistically significant using two-tailed comparisons. All experiments were repeated and analyzed independently.

## Results

### Inhibitory effects of Ssp(A4K-A11K) on bacterial adherence to experimental pellicles

**Fig 1:**
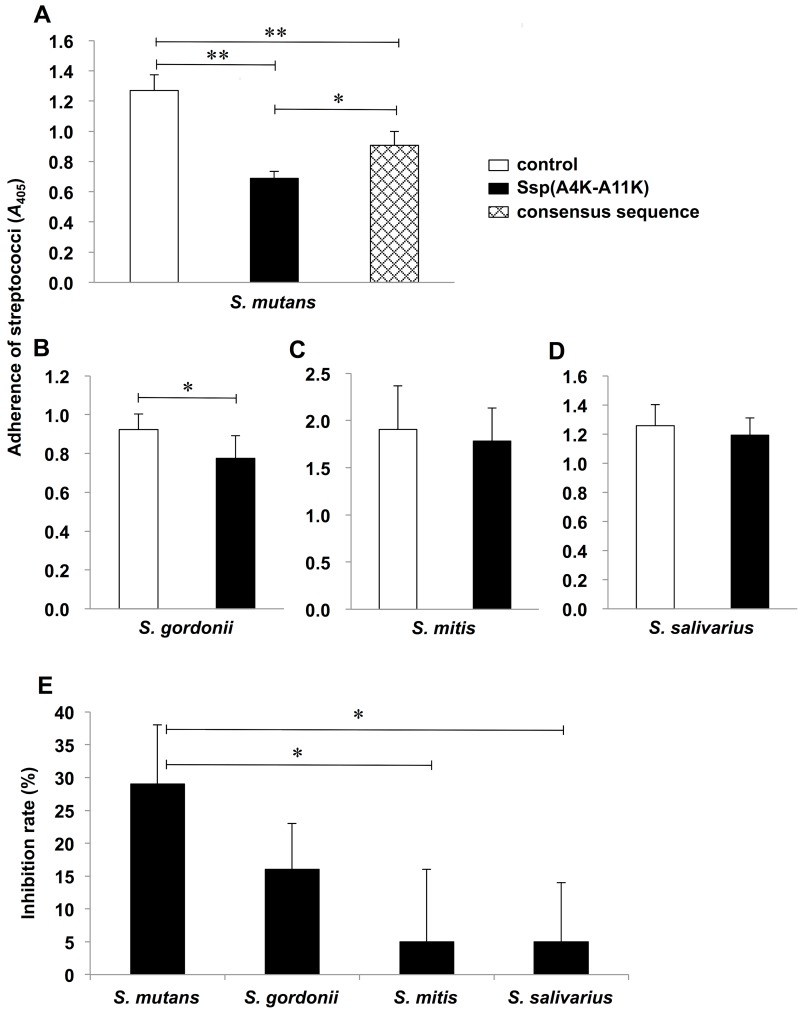
Effect of Ssp(A4K-A11K) on streptococcal adherence to saliva-coated surfaces. (A) *S*. *mutans*. (B) *S*. *gordonii*. (C) *S*. *mitis*. (D) *S*. *salivarius*. (E) Inhibition rate of streptococcal adherence to saliva-coated surfaces. Data are expressed as mean absorbance at 405 nm ± standard deviations (SDs) of three independent experiments with technical replicates. Asterisks denote significant differences (control: DW; * *P* < 0.05, ** *P* < 0.01).

To examine whether Ssp(A4K-A11K) is a potential inhibitor of *S*. *mutans* adherence to salivary pellicles; we performed inhibition assays using saliva-coated polystyrene plates (s-PS) (Fig 1). The SspA/B consensus sequence peptide showed anti-adherence activity against *S*. *mutans*, but the observed activity was significantly lower than that of Ssp(A4K-A11K) (Fig 1A). Adherence of *S*. *mutans* and *S*. *gordonii* were significantly inhibited by Ssp(A4K-A11K), whereas adherence of *S*. *mitis* and *S*. *salivarius* were not inhibited (Fig 1A–1D). Inhibition from strongest to weakest was *S*. *mutans* > *S*. *gordonii* > *S*. *mitis* ≈ *S*. *salivarius*. (Fig 1E). At peptide concentrations of 1,300 μM, the inhibitory effects on bacterial adherence were comparable to those at 650 μM (S1 Fig). According to previous studies [16, 17, 22], the reasonable peptide concentrations of SspA/B peptides range from 625 to 650 μM. Thus, a concentration of 650 μM of Ssp(A4K-A11K) was used for further studies.

### Inhibitory effects of Ssp(A4K-A11K) on *S*. *mutans* adherence to salivary agglutinin peptide SRCRP2

**Fig 2:**
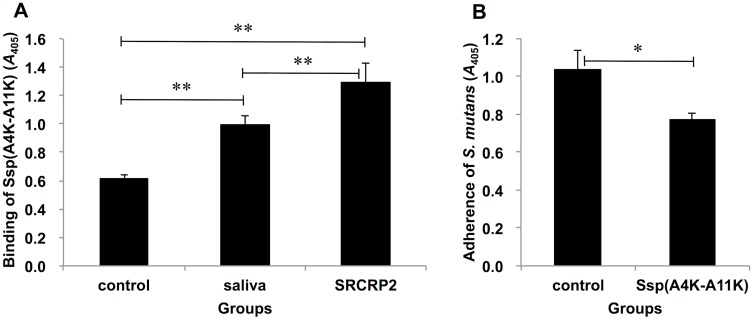
Effect of Ssp(A4K-A11K) on *S*. *mutans* adherence to SRCRP2. (A) Binding of Ssp(A4K-A11K) to SRCRP2. (B) Inhibition by Ssp(A4K-A11K) of *S*. *mutans* adherence to SRCRP2. Data are expressed as mean absorbance at 405 nm ± SDs of three independent experiments with technical replicates [control: (A) BSA or (B) DW; * *P* < 0.05, ** *P* < 0.01].

Salivary gp340 peptide SRCRP2 has been reported to have high binding activities to various streptococci [27]. We previously reported that Ssp(A4K-A11K) has binding activity with saliva, and furthermore, that salivary gp340 plays a key role in *S*. *mutans* adherence to polystyrene surfaces [11]. Thus, we performed a peptide inhibition assay where salivary gp340 peptide SRCRP2 was adsorbed onto 96-well polystyrene plates (Fig 2A). When wells were coated with 100 μl of SRCRP2 peptide (200 μg/ml), the binding of Ssp(A4K-A11K) peptide increased markedly (2-fold) compared to binding to wells without adsorbed SRCRP2 (Fig 2A). When wells were incubated with 100 μl of whole saliva, the binding by SspA/B peptide was significantly higher than in non-treated wells; however, binding was lower than that in SRCRP2-adsorbed wells (Fig 2A). In addition, the *S*. *mutans*-SRCRP2 interaction was significantly inhibited by Ssp(A4K-A11K) (Fig 2B), suggesting that salivary components, particularly gp340, promote adherence of *S*. *mutans* to polystyrene surfaces.

### Effects of Ssp(A4K-A11K) on *S*. *mutans* growth

**Fig 3:**
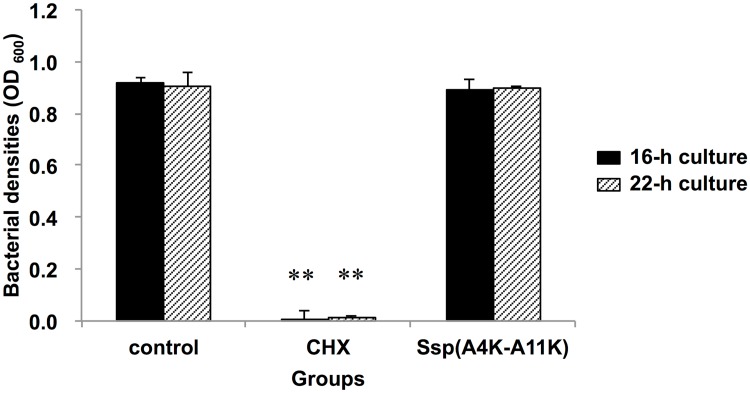
Bacterial growth in the presence of Ssp(A4K-A11K) or CHX. Ssp(A4K-A11K) or 0.04% CHX (a positive control) were applied to *S*. *mutans* cultures grown in BHI. The mixed suspensions were incubated for 16 or 22 h followed by measurement of the optical densities at 600 nm. Data are expressed as the means ± SDs of three independent assays (vs. control: PBS-treated *S*. *mutans*; ** *P* < 0.01).

A number of anti-microbial peptides form cationic amphipathic secondary structures, typically α-helices and β-sheets, that selectively interact with anionic bacterial membranes via electrostatic interactions [28]. Ssp(A4K-A11K), which includes multiple cationic amino acids, forms α-helical structures [22]; to determine whether the peptide has cationic anti-microbial activity, we performed a bactericidality assay. Growth of *S*. *mutans* cultures in BHI was significantly inhibited by chlorhexidine (0.04%) (*P* < 0.01), whereas the peptide (650 μM) did not affect bacterial growth, suggesting that the Ssp(A4K-A11K) does not have bactericidal activity at this concentration (Fig 3).

### Evaluation of biofilm inhibition by Ssp(A4K-A11K)

**Fig 4:**
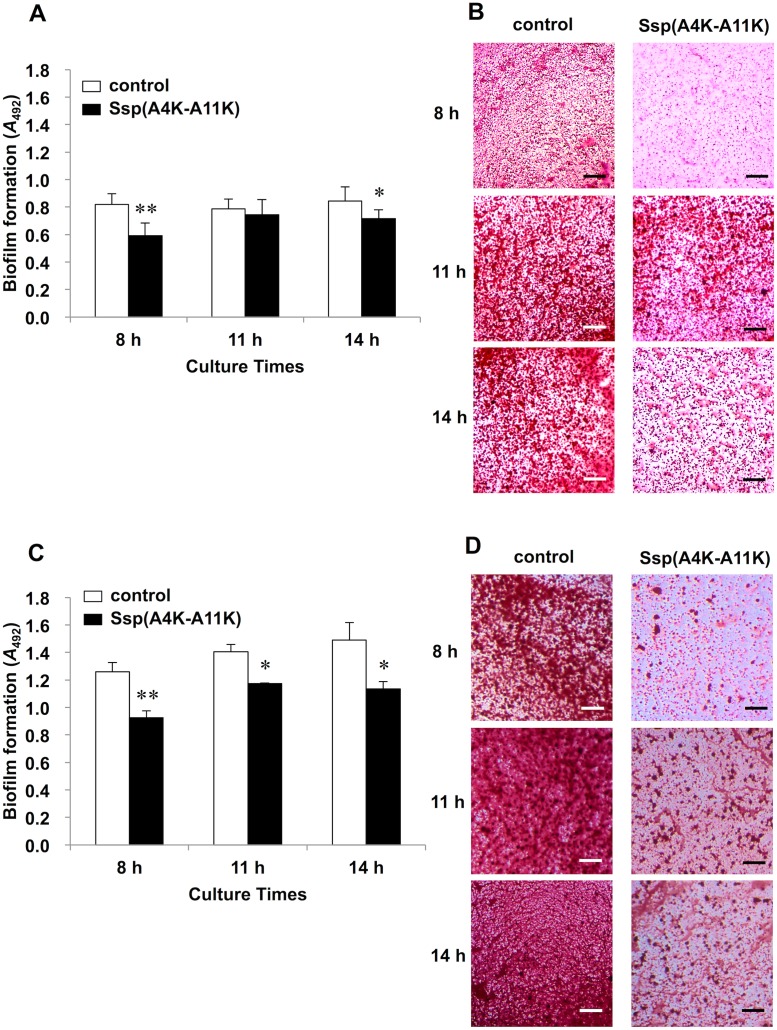
Inhibition using Ssp(A4K-A11K) for *S*. *mutans* biofilm formation on s-PS. (A) Biofilms formed during growth in TSBG. (B) Representative photographs of *S*. *mutans* biofilms in TSBG on s-PS at 8, 11, and 14 h culture (40×). Scale bars, 300 μm. (C) Biofilms formed during growth in TSBS. (D) Representative photographs of *S*. *mutans* biofilms in TSBS on s-PS at 8, 11, and 14 h culture (40×). Scale bars, 300 μm. Biofilms were stained with safranin and absorbance at 492 nm was measured. Data are expressed as the means ± SDs of three independent experiments with technical replicates (vs. control: non-treated s-PS; * *P* < 0.05, ** *P* < 0.01).

**Fig 5:**
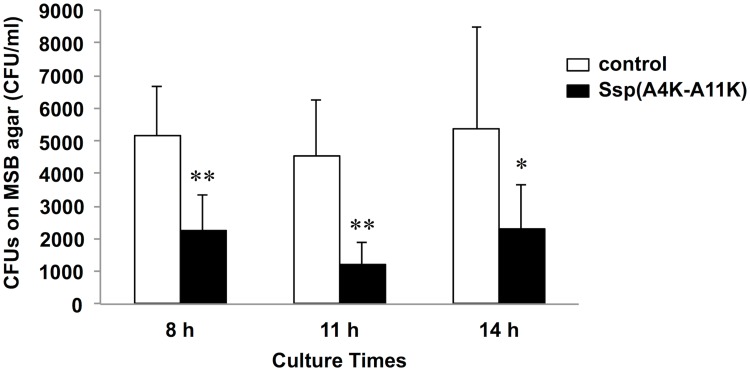
Inhibition using Ssp(A4K-A11K) for *S*. *mutans* biofilm formation on s-HA. The amounts of biofilms were measured by counting of colony-forming units (CFUs). Data are expressed as the means ± SDs of three independent experiments with technical replicates (vs. control: non-treated s-HA; * *P* < 0.05, ** *P* < 0.01).

**Fig 6:**
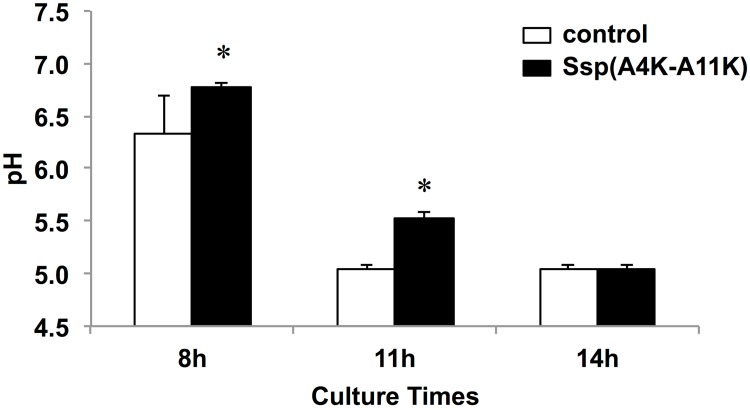
pH of culture media according to the time of biofilm formation. The pH of spent culture media was measured using a standard pH electrode. Data are expressed as the means ± SDs of three independent experiments with technical replicates (vs. control: non-treated s-PS; * *P* < 0.05).

As sessile populations reflect conditions in the state of nature more accurately than do planktonic bacteria, we next performed a biofilm formation assay. Koba et al. [22] have demonstrated that, compared to several analogous SspA/B peptides, the Ssp(A4K-A11K) peptide has the highest binding activity to salivary gp340 peptide SRCRP2 using the BIAcore assay. We previously demonstrated that Ssp(A4K-A11K) has binding activity to whole saliva in a peptide binding assay [11]. Hence, we hypothesized that Ssp(A4K-A11K) may inhibit *S*. *mutans* biofilm formation by competing for the same niche environment in the salivary pellicle. To assess this hypothesis, we examined *S*. *mutans* biofilm inhibition by using Ssp(A4K-A11K) (Fig 4). For biofilms grown in TSBG, pre-treatment with the SspA/B analogue peptide significantly diminished biofilm mass as compared with that of non-treatment groups at 8-h and 14-h culture times (Fig 4A). Inhibited biofilms on s-PS surfaces, stained in light color, were observed for Ssp(A4K-A11K) pre-treatment conditions at 8-h and 14-h culture times (Fig 4B). Sucrose is the most cariogenic dietary carbohydrate, and is used to produce the extracellular polysaccharides that form the biofilm matrix that facilitates the association of *S*. *mutans* with dental plaque. For biofilms grown in TSBS, pre-treatment with the Ssp(A4K-A11K) showed evident biofilm inhibition at all culture times (Fig 4C). Diminished biofilms on s-PS were observed for Ssp(A4K-A11K) pre-treatment conditions at all culture times (Fig 4D). To provide further confirmation, we evaluated bacterial population of biofilms on saliva-coated HA disks (s-HA) by detaching biofilms from s-HA, plating the resulting cell suspension on MSB agar, and counting the resulting CFUs (Fig 5). Pre-treatment with 650 μM analogue peptide markedly reduced CFU counts compared to non-treatment at all culture times (*P* < 0.01) (Fig 5). Because the metabolic activity of the bacteria leads to acidification of the milieu, changes in pH values over time were analyzed to estimate biofilm acidogenicity. The data showed significantly higher pH values in Ssp-treated biofilms than in non-treatment groups at 8-h and 11-h culture times (*P* < 0.05) (Fig 6).

## Discussion

Oral infectious diseases can be prevented by controlling oral biofilm microorganisms. Sessile organisms in biofilms are more resistant to anti-microbial agents, host defenses, and stress than cells in the planktonic state, making sessile organisms more difficult to control. The first step in biofilm development is microbial adherence to the host; therefore, we focused on an anti-adherence strategy that could inhibit a crucial step of biofilm formation. As for preventing *S*. *mutans* from adhering to salivary pellicles, promising outcomes have been obtained by using peptides that mimic the streptococcal adhesin AgI/II (p1025) [18] and SspB [11, 17]; phosphoproteins and phosphoprotein-like substances that hydrophilize the tooth surface, such as statherin [28], histatin 1 [29], and phosphorylated polyethylene glycol (PEG) [19]; and prereacted glass ionomer (S-PRG) eluate [20]. However, the safety (impact on resident oral microflora), specificity (targeting specific pathogen), and stability of such anti-adherence agents require further evaluation. In the present study, we investigated the inhibitory effects of Ssp(A4K-A11K) on adherence and biofilm formation of *S*. *mutans*; we also evaluated relevant properties (including bactericidality, specificity, and duration) of the peptide.

In the present work, we demonstrated that Ssp(A4K-A11K) effectively prevents biofilm formation by *S*. *mutans* (Figs 4 and 5). When the wells were pre-treated with the SspA/B analogue peptide, diminished biofilm formation was observed at 8 h and 14 h culture (but not at 11 h) in TSBG (Fig 4). On the other hand, biofilm formation grown in glucan-inducing media including 0.25% sucrose (TSBS) was markedly inhibited by pre-treatment with the Ssp(A4K-A11K) at all culture times (Fig 4C–4D). The ability of *S*. *mutans* to metabolize sucrose using glucosyltransferase enzymes (Gtfs) is clinically relevant to caries development. *S*. *mutans* readily converts dietary sucrose to insoluble glucans, which in turn promote adhesion of *S*. *mutans* to the tooth surface, leading to the formation of highly adhesive and cohesive biofilms [30–32]. In our work, distinct biofilm inhibition was observed in TSBS compared with that in TSBG (Fig 4). Presumably, the observed differences in the results obtained with TSBS and TSBG indicate that once bacterial adherence is inhibited by Ssp(A4K-A11K), biofilm formation is prevented, despite the presence of sucrose in the culture medium; hence, differences between peptide-treated and non-treated biofilm mass in TSBS may be enhanced by the volume of sucrose-derived insoluble glucans. It may be the reason why the 11-h time point observed in TSBG does not appear to give the same results as the flanking time points. To provide further confirmation, we evaluated the anti-biofilm activity of Ssp(A4K-A11K) against biofilms on s-HA by detaching biofilms from s-HA, plating the resulting cell suspensions to MSB agar, and enumerating CFUs. Pre-treatment with 650 μM analogue peptide dramatically reduced CFU counts compared to non-treatment at all culture times (Fig 5), indicating that the formation of biofilms on s-HA was inhibited by Ssp(A4K-A11K). The results therefore suggest that Ssp(A4K-A11K) may be efficacious in combatting early stage cariogenic biofilms formed on pellicle-covered teeth. For impacts of Ssp analogue to later stage biofilm, inhibition was found only in 48-h biofilms grown in TSBS (S3 Fig). Although the cause behind this inhibition is still unclear, it could be explained, in part, by incomplete biofilm development in later stage where nutrients are exhausted. This 96-well microtiter biofilm assay uses static, batch-growth conditions and it probably does not allow for the formation of the mature biofilms typically associated with continuous cultivation. Thus, future work should focus on the impact of Ssp analogue to later stage biofilm by using continuous cultivation systems such as flow chamber experiments.

Acidogenicity of *S*. *mutans* may explain the distinct cariogenic potentials. Kim et al. [33] have demonstrated that cranberry-derived flavonoids (CranFlav) inhibit *S*. *mutans* biofilm formation with elevated pH values at the biofilm/sHA interface compared to vehicle-treated biofilms, being in agreement with our data (Fig 6). The critical pH, below which enamel demineralization begins, is recently reported to be as low as 5.1 [34]. Notably, pH in Ssp-treated 8-h (6.8 ± 0.06) and 11-h (5.5 ± 0.06) biofilms showed higher values than the critical pH (Fig 6). This could be explained that Ssp(A4K-A11K) interfered with *S*. *mutans* adherence, resulting in the delay of pH reduction.

Many anti-bacterial peptides are known to kill bacteria by permeabilizing and/or disrupting bacterial membranes via electrostatic interactions between cationic amphipathic α-helices and anionic bacterial membranes [28]. Intriguingly, Ssp(A4K-A11K) includes cationic amino acid residues and forms α-helical structures [22]. Consequently, we performed a bactericidal assay to determine whether Ssp(A4K-A11K) has bactericidal activity (Fig 3). Notably, treatment with 650 μM of Ssp(A4K-A11K) did not show bactericidal effect against planktonic cells, implying that the anti-biofilm activities are attributable to reduced bacterial adherence to the experimental pellicles. Therefore, the use of this peptide may permit control of cariogenic biofilm formation without the risk of unfavorable disruption of the dynamic balance between the host and the resident oral microflora.

The streptococcal peptide analogue Ssp(A4K-A11K) was designed by double-lysine substitution in the consensus sequence of the SspA/B peptides (Table 1). Koba et al. showed that the distance between the positively charged amino acids K4 and K11 in the Ssp(A4K-A11K) was similar to that between negatively charged amino acids Q1and E5 in SRCRP2 [22]. The position of the lysine has been considered to be an important factor for binding of the SspA/B peptide to both the negatively charged salivary components [16, 17] and to the negative charges in the SRCRP2 β-sheet [22]. Additionally, ion-pairing interactions between oppositely charged amino acids (lysine and negatively charged functional groups) are important for protein structural stability [21]. The residue immediately C-terminal to the lysine at position 11 is the negatively charged glutamic acid at position 12 in the Ssp(A4K-A11K) (Table 1). Okuda et al. [17] reported that the lack of glutamic acid adjacent to lysine-11 resulted in a significant loss of the inhibitory activity for the SspB peptide. Hence, the position of the charged amino acid residues in the peptide is essential for the peptide’s binding activity, adherence inhibition, and peptide stability. The anti-biofilm activity of SspB(390-T400K-402) was actually limited to a short culture time (within 8 hours) [17], whereas Ssp(A4K-A11K) exhibited prolonged activity (until 14 hours) in both TSBG and TSBS in the present study. Indeed, in our preliminary study we found that SspB(390-T400K-402) did not exert significant anti-biofilm activity at 14 h (S2 Fig). This distinction probably reflects improved peptide stability by double-lysine substitution in the consensus sequence of SspA/B peptides, permitting the formation of stable ion-parings. We believe these data to be clinically relevant because inhibitory effect that sustains for 14 hours is sufficient to keep oral health in combination with oral hygiene routine including regular brushing and flossing, and limiting sugar intake.

**Table 1:** Amino acid sequences of SspA/B peptides.

Peptide	Amino acid sequence
Consensus sequence	^1^D Y Q A K L A A Y Q A E L^13^^a^
Ssp (A4K-A11K)	D Y Q **K**^b^ K L A A Y Q **K** E L

^a^Number indicated position in 13 mer amino acids residues.

^b^The substituted amino acid with lysine was indicated in bold.

Here, we showed preferential adherence inhibition of *S*. *mutans* on s-PS as compared with commensal streptococci (*Streptococcus mitis* and *Streptococcus salivarius*) (Fig 1). For clinical application, anti-plaque agents need to be harmless to beneficial commensals, given that resident streptococci are thought to contribute to the stability of the oral community [35, 36]. The anti-adherence effects were observed in the bacterial adherence assay with *S*. *mutans* but not with *S*. *mitis* or *S*. *salivarius* (Fig 1A, 1C and 1D). The inhibitory effects were also observed with *S*. *gordonii* (Fig 1B), although the degree of inhibition was less than that observed with *S*. *mutans* (Fig 1E); however, that result differs from the outcomes of a previous study [17]. Our results demonstrate that the anti-adherence activity of Ssp(A4K-A11K) is superior to that of the SspA/B consensus sequence peptide (Fig 1A). The lysine substitution incorporated into Ssp(A4K-A11K) is expected to yield conformational changes to the peptide, which may shed light on the altered peptide properties observed in the present work (Fig 1A and 1E). Preliminary experiments have also shown that Ssp(A4K-A11K) is innocuous in an inhibition of other streptococcal biofilm formed by *S*. *gordonii*, *S*. *mitis*, or *S*. *salivarius* (S4 Fig). As far as the diminished inhibitory effects against *S*. *gordonii* is concerned, two possibilities can be considered. The first is conformational changes in Ssp(A4K-A11K), resulting from double-lysine substitution in the consensus sequence of the SspA/B peptides, have some effects on its characteristics. The second is interference by the other adhesin of *S*. *gordonii*, Hsa, a sialic acid binding polypeptide [10]. Pellicle receptors for Hsa include not only gp340 but also mucin MG2 and the sIgA heavy chain [15]; thus, blocking of *S*. *gordonii*-gp340 interaction by Ssp(A4K-A11K) could be insufficient for adherence and biofilm inhibition. As for *S*. *mitis* and *S*. *salivarius*, they have different adhesins, which have binding function to α-amylase or extra parotid glycoprotein in saliva [37], not to gp340. Therefore, *S*. *mitis* and *S*. *salivarius* were not affected by Ssp(A4K-A11K) pre-incubation both in adhesion and biofilm formation. These findings, considered in combination with our other results (Fig 3), suggest that Ssp(A4K-A11K) may be a significant and specific inhibitor for the adherence of *S*. *mutans* to saliva-coated surfaces.

Pre-coating of well with the salivary gp340 peptide SRCRP2 (200 μg/ml) yielded enhanced binding by Ssp(A4K-A11K) (Fig 2A), consistent with previous work [22]. Our experiments also demonstrated that pre-treatment of Ssp(A4K-A11K) inhibited *S*. *mutans* adherence to SRCRP2 (Fig 2B). These results indicated that Ssp(A4K-A11K) has binding affinity for salivary gp340, thereby competitively blocking the *S*. *mutans*-salivary pellicle interaction.

Collectively, these results suggest that the inhibitory effects of Ssp(A4K-A11K) on *S*. *mutans* biofilms observed here are due to competitive inhibition of adherence of the bacteria to salivary gp340. Further studies will be required to examine whether this strategy can be effective in protecting hosts from cariogenic biofilm maturation and caries development *in vivo*. Notably, NOD/SCID.*e2f1-/-*, a candidate humanized mouse model, may be suitable for assessing prophylaxis against dental biofilm-dependent diseases such as dental caries [38]. In conclusion, we used *in vitro* assays to demonstrate that Ssp(A4K-A11K) exhibits improved safety, specificity, and stability in inhibiting *S*. *mutans* biofilm formation compared to the parent consensus SspA/B peptide. These findings may be considered complementary to research into anti-plaque agents.

## Supporting information

S1 Fig Effect of Ssp(A4K-A11K) at 1,300 μM on *S*. *mutans* adherence to saliva-coated surfaces. Data are expressed as mean absorbance at 405 nm ± SDs of three independent experiments with technical replicates Asterisks denote significant differences (control: DW; * *P* < 0.05, ** *P* < 0.01).
(TIFF)

S2 Fig Effect of SspB(390-T400K-402) on 14-h *S*. *mutans* biofilm formation on s-PS. (A) Biofilms formed during growth by cultures pre-treated with SspB(390-T400K-402). (B) Representative photographs of *S*. *mutans* biofilms in TSBS on s-PS at 14 h of culturing (40×). Scale bars, 300 μm. Biofilms were stained with safranin and the absorbance at 492 nm was measured. Data are indicated relative to the biofilm formation observed in control, set = 1.0. Values are expressed as means ± SDs of three independent experiments with technical replicates.
(TIFF)

S3 Fig Effect of Ssp(A4K-A11K) on later stage biofilm formation of *S*. *mutans*. (A) Biofilms formed during growth in TSBG. (B) Representative photographs of *S*. *mutans* biofilms in TSBG on s-PS at 48 h, 72 h, and 1 week culture (40×). Scale bars, 300 μm. (C) Biofilms formed during growth in TSBS. (D) Representative photographs of *S*. *mutans* biofilms in TSBS on s-PS at 48 h, 72 h, and 1 week culture (40×). Scale bars, 300 μm. Biofilms were stained with safranin and absorbance at 492 nm was measured. Data are expressed as the means ± SDs of three independent experiments with technical replicates (vs. control: non-treated s-PS; * *P* < 0.05).
(TIFF)

S4 Fig Effect of Ssp(A4K-A11K) on other streptococcal biofilm formation in TSBS on s-PS. Biofilms formed during growth by cultures pre-treated with Ssp(A4K-A11K) (left). Representative photographs of streptococcus biofilms in TSBG on s-PS at 8, 11, and 14 h culturing (40×) (right). Scale bars, 300 μm. Biofilms were stained with safranin and the absorbance at 492 nm was measured. Data are expressed as the means ± SDs of three independent experiments with technical replicates.
(TIFF)
