# Supplementary figures and images for: Streptococcal adhesin SspA/B analogue peptide inhibits adherence and impacts biofilm formation of Streptococcus mutans
Source: PLoS One. 2017 Apr 10;12(4):e0175483. doi: 10.1371/journal.pone.0175483 (PMC5386287; doi:10.1371/journal.pone.0175483)

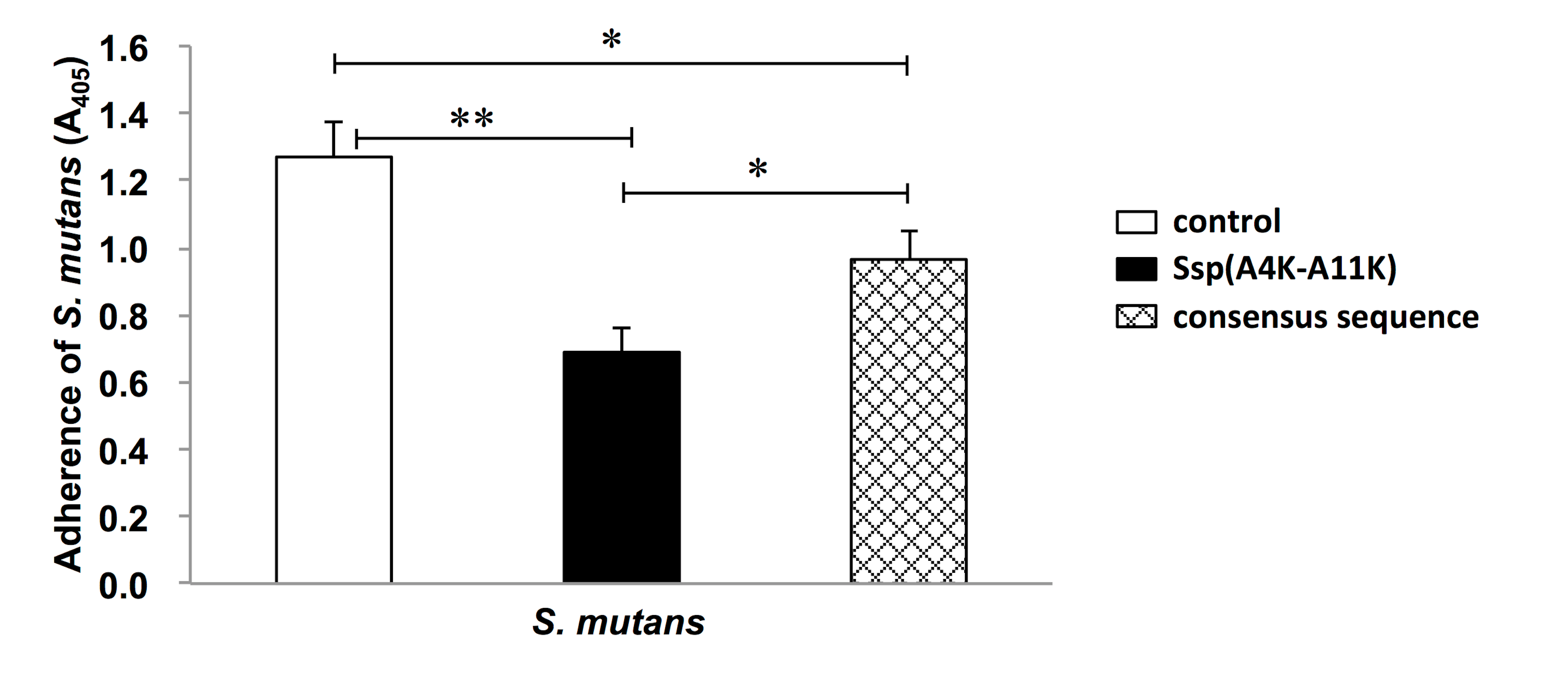

Supplement: S1 Fig — Data are expressed as mean absorbance at 405 nm ± SDs of three independent experiments with technical replicates Asterisks denote significant differences (control: DW; * P < 0.05, ** P < 0.01). (TIFF) [file pone.0175483.s001.tiff]

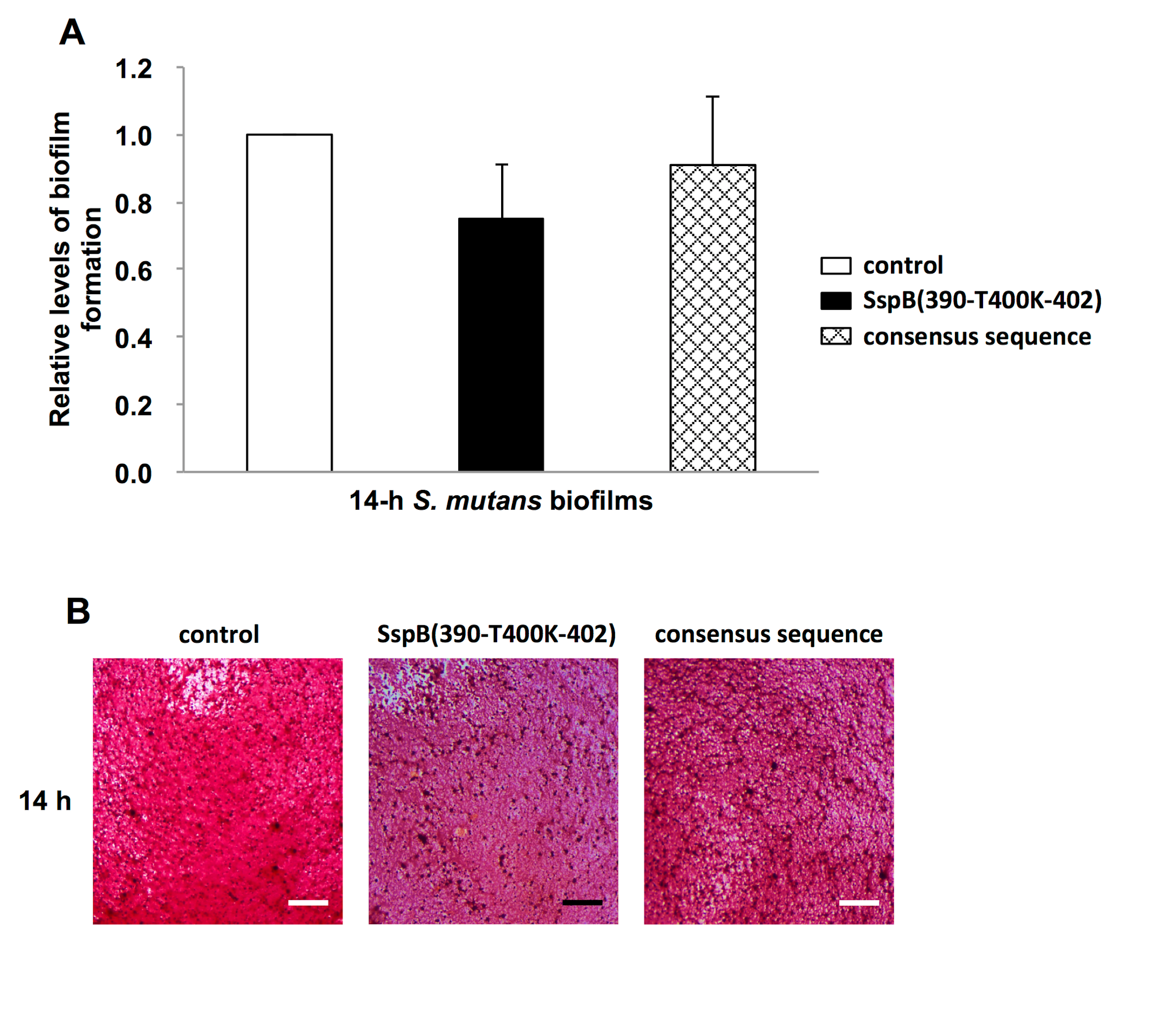

Supplement: S2 Fig — (A) Biofilms formed during growth by cultures pre-treated with SspB(390-T400K-402). (B) Representative photographs of S. mutans biofilms in TSBS on s-PS at 14 h of culturing (40×). Scale bars, 300 μm. Biofilms were stained with safranin and the absorbance at 492 nm was measured. Data are indicated relative to the biofilm formation observed in control, set = 1.0. Values are expressed as means ± SDs of three independent experiments with technical replicates. (TIFF) [file pone.0175483.s002.tiff]

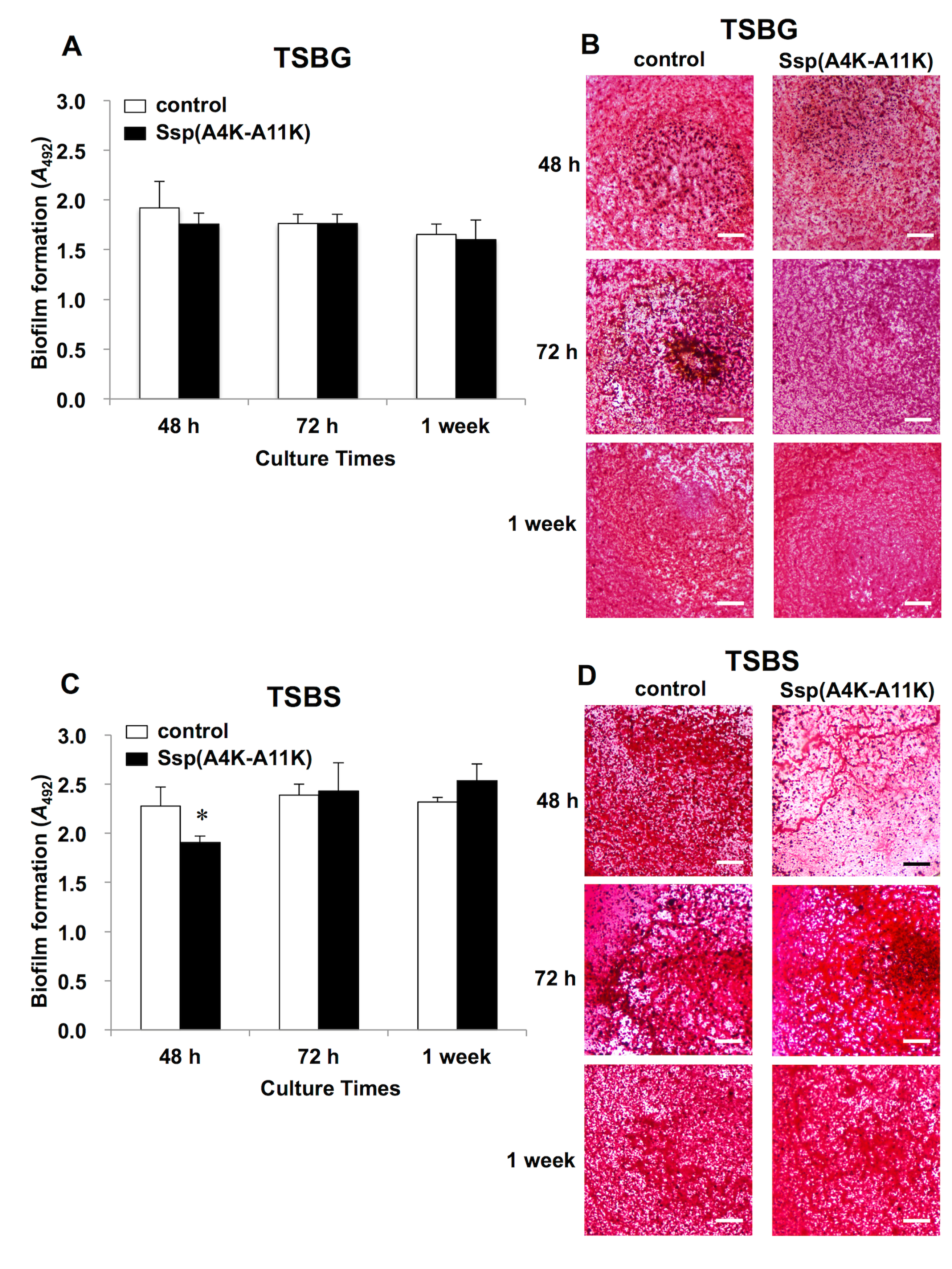

Supplement: S3 Fig — (A) Biofilms formed during growth in TSBG. (B) Representative photographs of S. mutans biofilms in TSBG on s-PS at 48 h, 72 h, and 1 week culture (40×). Scale bars, 300 μm. (C) Biofilms formed during growth in TSBS. (D) Representative photographs of S. mutans biofilms in TSBS on s-PS at 48 h, 72 h, and 1 week culture (40×). Scale bars, 300 μm. Biofilms were stained with safranin and absorbance at 492 nm was measured. Data are expressed as the means ± SDs of three independent experiments with technical replicates (vs. control: non-treated s-PS; * P < 0.05). (TIFF) [file pone.0175483.s003.tiff]

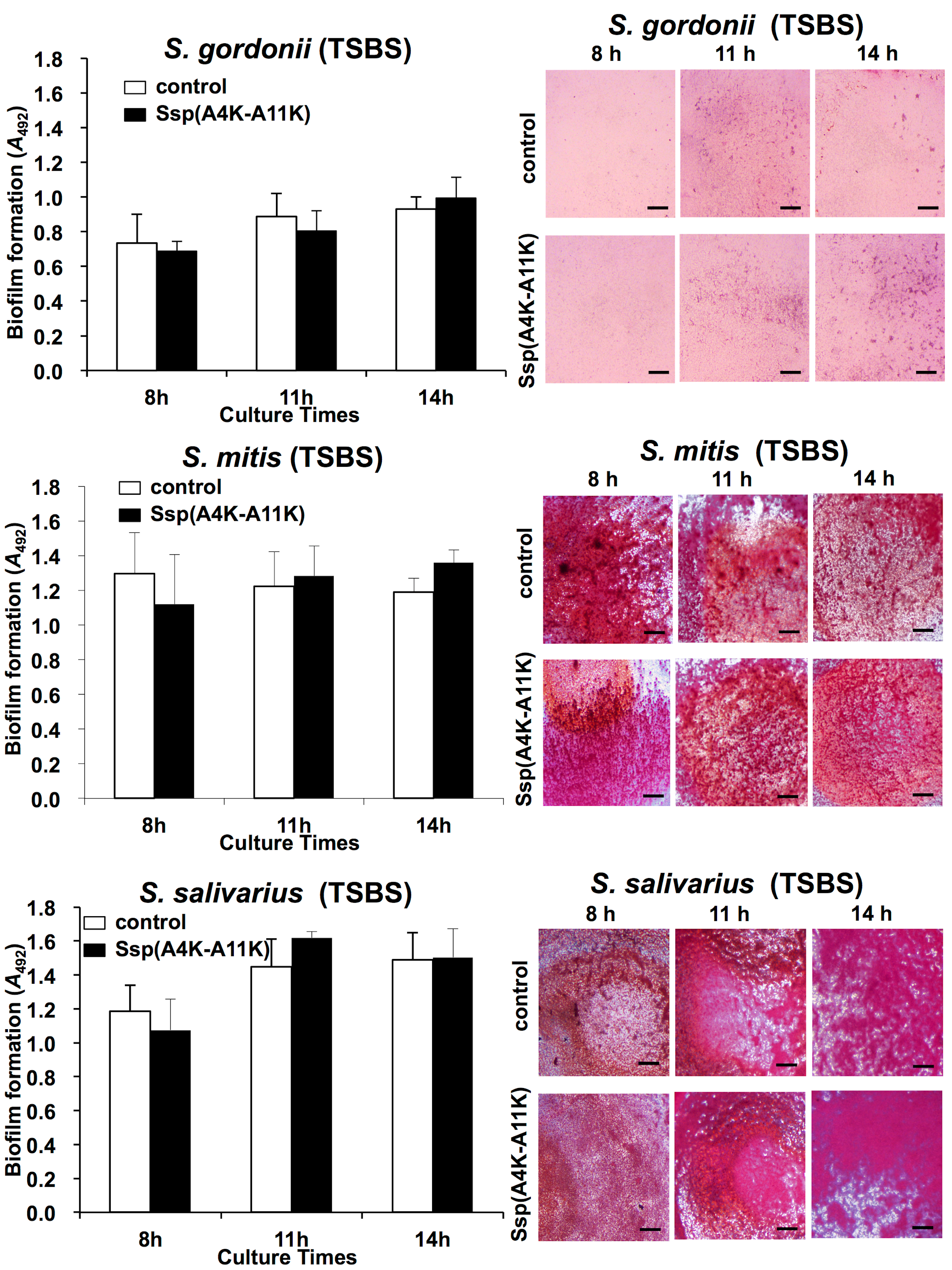

Supplement: S4 Fig — Biofilms formed during growth by cultures pre-treated with Ssp(A4K-A11K) (left). Representative photographs of streptococcus biofilms in TSBG on s-PS at 8, 11, and 14 h culturing (40×) (right). Scale bars, 300 μm. Biofilms were stained with safranin and the absorbance at 492 nm was measured. Data are expressed as the means ± SDs of three independent experiments with technical replicates. (TIFF) [file pone.0175483.s004.tiff]
